# Improved Cycling Stability of LiCoO_2_ at 4.5 V via Surface Modification of Electrodes with Conductive Amorphous LLTO Thin Film

**DOI:** 10.1186/s11671-020-03335-8

**Published:** 2020-05-14

**Authors:** Shipai Song, Xiang Peng, Kai Huang, Hao Zhang, Fang Wu, Yong Xiang, Xiaokun Zhang

**Affiliations:** 1 School of Materials and Energy, University of Electronic Science and Technology of China, Chengdu, 611731 Sichuan China; 2 Advanced Energy Research Institute, University of Electronic Science and Technology of China, Chengdu, 611731 Sichuan China

**Keywords:** Lithium ion batteries, LiCoO_2_, High voltage, Cycling stability, Surface modification

## Abstract

The stability issue of LiCoO_2_ cycled at high voltages is one of the burning questions for the development of lithium ion batteries with high energy density and long cycling life. Although it is effective to improve the cycling performance of LiCoO_2_ via coating individual LiCoO_2_ particles with another metal oxides or fluorides, the rate capacity is generally compromised because the typical coating materials are poor conductors. Herein, amorphous Li_0.33_La_0.56_TiO_3_, one of the most successful solid electrolytes, was directly deposited on the surface of made-up LiCoO_2_ electrodes through magnetron sputtering. Not only the inherent conductive network in the made-up LiCoO_2_ electrodes was retained, but also the Li^+^ transport in bulk and across the cathode-electrolyte interface was enhanced. In addition, the surface chemical analysis of the cycled LiCoO_2_ electrodes suggests that most of the stability issues can be addressed via the deposition of amorphous Li_0.33_La_0.56_TiO_3_. With an optimized deposition time, the LiCoO_2_ electrodes modified by Li_0.33_La_0.56_TiO_3_ performed a steady reversible capacity of 150 mAh/g at 0.2 C with the cutoff voltage from 2.75 to 4.5 V vs*.* Li^+^/Li and an 84.6% capacity gain at 5 C comparing with the pristine one.

## Introduction

Lithium ion batteries (LIBs) have been urged for high energy density, high rate capability, and long cycling life, with increasing energy storage demands in portable electronics, electrical vehicles, and stationary power sources [1–3]. The most direct way to increase LIBs’ energy density is to apply cathode materials with higher capacities and/or higher working voltages [4–8]. LIBs with LiCoO_2_ (LCO) cathode has gained great commercial success in the past 3 decades, especially as the power source for portable electronics, benefiting from its high specific capacity, high redox potential, and long cycling life [9–12]. However, the generally utilized specific capacity of LCO can only reach 140 mAh/g, roughly half of its theoretical capacity of 272 mAh/g, with the upper cutoff voltage of 4.2 V vs. Li^+^/Li [11–13]. Theoretically, the utilized specific capacity can be improved by increasing the cutoff voltage. However, the cycling stability of LCO is poor when the cutoff voltage exceeds 4.2 V vs. Li^+^/Li [1]. In addition, it is demonstrated that the capacity decay of LCO below 4.5 V vs. Li^+^/Li is mainly due to the cacoethic side reactions, Co dissolution, and HF corrosion at the liquid-solid interface between LiPF_6_-based organic electrolyte and LCO cathode [14, 15]. Therefore, great efforts have been made to realize a stable cathode-electrolyte interface at 4.5 V vs. Li^+^/Li via surface modifications of LCO [16–18].

In terms of structural feature, the surface modifications can be divided into two types. In one type, the modification layer is coated on individual LCO particles before casting the electrodes [16–18]. In the other type, the modification layer is deposited on the surface of made-up LCO electrodes [19, 20]. Although the surface modification on individual LCO particles is effective to improve its cycling stability [16–18], and can be easily realized via low-cost wet chemical routes [21–24], there are some disadvantages that limit its application. For example, the modification layer on particles may break due to the severe mechanical impacts during slurry mixing and electrode calendering [13]. In addition, the modification layer on individual particles may tip the balance of ionic conductivity and electronic conductivity in the bulk of electrodes [1]. Alternatively, the surface modification of made-up LCO electrodes, which is carried out after LCO granulating, slurry mixing, and electrode calendaring, and only introduces a thin layer of modification materials on the surface of electrodes, is potential to addressing the above issues [13, 19, 20, 25].

In terms of material chemistry, the surface modifications can be realized by inert compounds including fluorides (AlF_3_, CeF_3_, LaF_3_, etc.) [21, 22] and oxides (Al_2_O_3_, MgO, ZrO_2_, ZnO, etc.) [23–26], which are generally poor Li^+^ and e^−^ conductors or ionic conductors for Li^+^ (LiAiO_2_, Li_4_Ti_5_O_12_, Li_3_PO_4_, Li_2_CO_3_, etc.) [13, 19, 20, 27]. Although the surface modifications by inert compounds are helpful to stabilize the LCO-electrolyte interface at high voltages [27], they may cripple the rate capability of LCO cathode, since the charge transport and transfer would be limited by the low-conductive coating layer [19, 27]. On the other hand, the surface modifications by Li^+^ conductors would not attenuate the bulk conductive network in cathode significantly, while the interfacial stability can be improved [20, 25]. Especially, a Li^+^-conductive interfacial layer would help the Li^+^ migration between LiPF_6_-based electrolyte and LCO cathode, resulting in a desired small interfacial impedance [28].

**Fig. 1:**
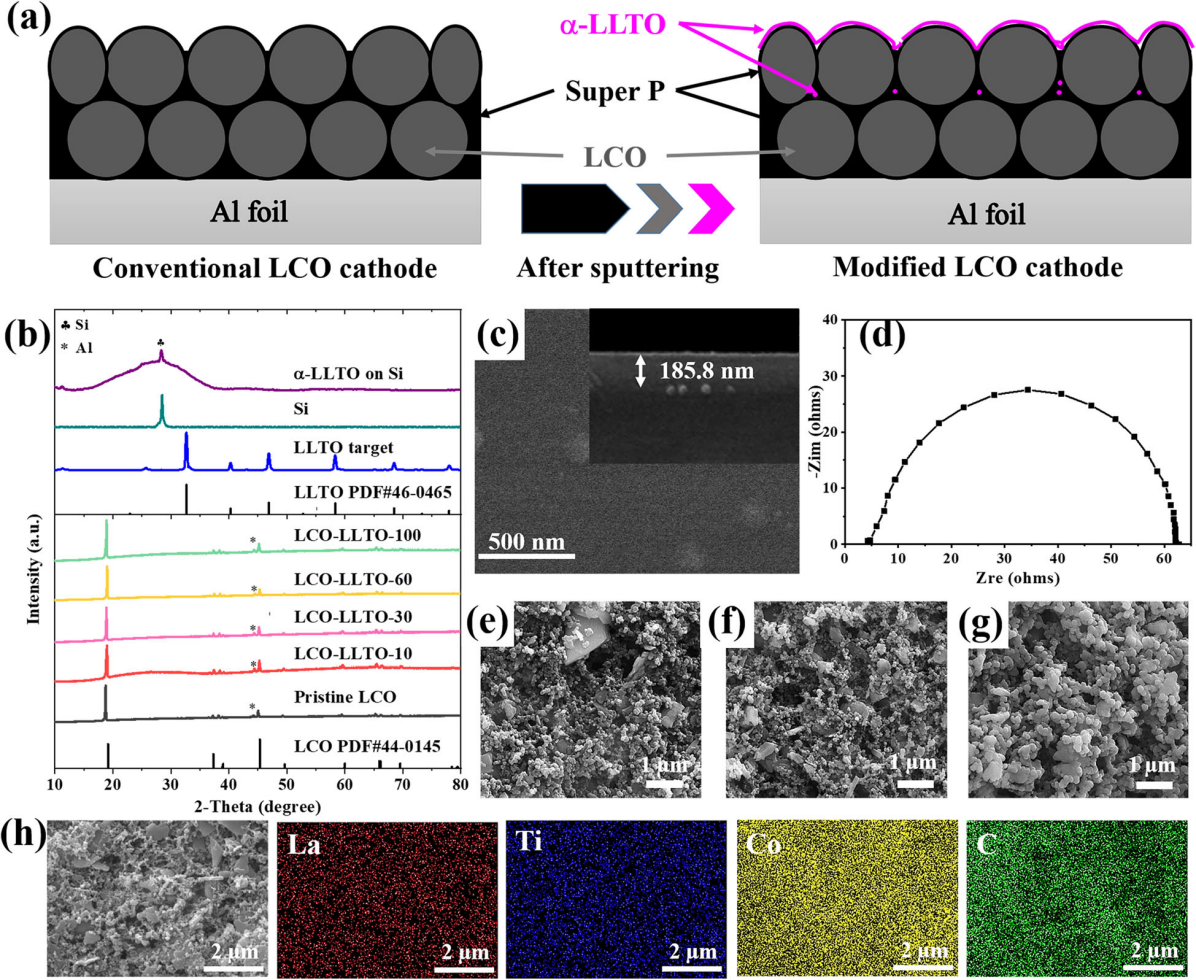
**a** Schematic illustration of conventional LCO electrode and LCO electrode with an α-LLTO modification layer; **b** XRD patterns of LLTO target (blue line), Si substrate (dark-cyan line), α-LLTO thin-film on Si substrate (purple line), pristine LCO cathode (black line), and α-LLTO-modified LCO electrodes with different deposition time of LLTO (red line for 10 min, pink line for 30 min, yellow line for 60 min, and green line for 100 min); **c** Top- and side-view SEM images of LLTO thin-film on Si substrate; **d** EIS cure of LLTO film on Si substrate; **e**–**g** Top-view SEM images of **e** pristine LCO, **f** LCO-LLTO-10, and **g** LCO-LLTO-100; **h** EDS mapping of the surface of LCO-LLTO-10

Herein, amorphous Li_0.35_La_0.56_TiO_3_ (α-LLTO), which is one of the most successful solid electrolytes [29, 30], was directly deposited on the surface of made-up LCO electrodes through magnetron sputtering (Fig. 1a). The sputter-deposited α-LLTO does not require high temperature heat treatment and performs a high ionic conductivity (1.54 × 10^−5^ S/cm at room temperature). It is inspiring that the electrode level surface modification by α-LLTO not only does not impair the bulk conduction in LCO cathode, but also enhances the charge transfer kinetics at LCO-electrolyte interface, which is favorable for rate capacity. In addition, the deposited α-LLTO effectively prevents Co dissolution, HF corrosion, and other side reactions at LCO-electrolyte interface. The LCO-LLTO-electrolyte configuration leads to a relatively stable interfacial polarization. As a result, the presented surface modification of electrodes with α-LLTO enables LCO steady operates for more than 100 cycles with an upper cutoff voltage of 4.5 V vs*.* Li^+^/Li and a reversible capacity of 150 mAh/g at 0.2 C.

## Material and Methods

### The Preparation of LCO Cathode and Surface Modification by α-LLTO

LCO electrodes were prepared by spreading well-mixed commercial LCO powders (Aladdin, ~ 2 μm, 99%, 80 wt%), acetylene black (MTI KJ Group, 10 wt%), and PVDF (Arkema, 10 wt%) on the surface of Al foil. N-methyl-pyrrolidone was used as solvent to form the slurry. The as-casted LCO electrodes were dried in dynamic vacuum overnight at 110 °C to remove the solvent and trace water after calendering. The statistic thickness of the casted LCO cathode is ~ 40 μm, which is determined by a screw micrometer. The loading density of LiCoO_2_ active material is 4.96 mg/cm^2^ (0.97 mAh/cm^2^ for the first discharge from 4.5 to 2.75 V vs. Li/Li^+^). α-LLTO was deposited on the surface of Si substrates or LCO electrodes by magnetron sputtering. The cavity was evacuated to 5 × 10^−4^ Pa or less. The LCO electrodes were pre-heated at 120 °C for 30 min in vacuum to remove the trapped moisture and air. The Li_0.33_La_0.56_TiO_3_ target was pre-sputtered for 5 min to remove dust and foreign particles on the surface. The distance between target and substrate was 15 cm. The sputtering power was 120 W. The working pressure was 1 Pa. The argon and oxygen ratio was 70:30 (sccm). The substrate temperature was kept at 120 °C. To obtain the modification layers with different thickness, the sputtering time was set to 10, 30, 60, and 100 min. Samples with different α-LLTO deposition time were denoted as LCO-LLTO-10, LCO-LLTO-30, LCO-LLTO-60, and LCO-LLTO-100, respectively. After the deposition of α-LLTO, the obtained samples were dried under vacuum for 24 h to remove trapped moisture prior to use. To determine the ionic conductivity of α-LLTO, it was deposited on a flat Si substrate. The deposition process parameters were similar to the surface modification of LCO electrodes except for the deposition time that was 240 min.

### Material Characterizations

The thickness of LLTO films on Si substrate were determined using cross-sectional scanning electron microscopy (SEM). The phase analysis was performed by X-ray diffraction (XRD) using CuKα radiation. The surface morphology of LCO electrodes were observed by SEM. The elemental distributions of Co, C, La, and Ti were analyzed by Energy Dispersive Spectrometer (EDS). X-ray photoelectron spectroscopy (XPS, Thermo Fisher Escalab Xi+) was used to analyze the surface chemical compositions of the electrodes.

### Electrochemical Measurements

The LCO electrodes were punched into circles with a diameter of 12 mm and dried in dynamic vacuum overnight at 80°C to remove trace water absorbed from the air. The separators (polypropylene, Celgard 2400) were dried in vacuum overnight at 50 °C. The electrochemical properties of samples were tested in 2032 coin cells equipped with a lithium metal anode. The liquid electrolyte was 1 M LiPF_6_ solution with the mixed EC:DMC:EMC (v/v/v = 1:1:1) solvent. All the cells were fabricated in an Ar-filled glove box. Cycling tests were performed between 2.75 and 4.5 V vs. Li^+^/Li with varied charge-discharge rates at room temperature by the battery test equipment (NEWARE CT-3008). Cyclic voltammetry (CV) and electrochemical impedance spectroscopy (EIS) were carried out by Princeton VersaSTAT 3F electrochemical analyzer. For EIS measurements, the amplitude voltage was 10 mV, and the frequency range was 0.1 Hz to 100 kHz.

## Results and Discussion

Energy density and rate capability are the two core requirements for cathode technologies. It raises the challenge for the surface modification of LCO cathode. The conductive network in the electrode for Li^+^ and e^−^ should be maintained, while the LCO-electrolyte interface is stabilized via introducing inactive materials as little as possible. As shown in Fig. 1a, we propose coating the made-up LCO electrode with α-LLTO through magnetron sputtering. The sputter-deposited α-LLTO would form a conformal, dense, and very thin overburden on the surface of LCO electrode. The following merits can be reasonably expected. First, the potential damages to the modification layer during the preparing process of electrodes are avoided. Second, the mass fraction of α-LLTO in the modified electrode is very small. Third, the undesirable interactions between LCO and electrolyte can be suppressed effectively. Last and most importantly, the deposited α-LLTO will not undermine the transport pathways for Li^+^ and e^−^ in the electrode because it mainly exists near the top surface of the electrode.

The X-ray diffraction peaks derived from the Li_0.35_La_0.56_TiO_3_ target used here are well identical with crystalline LLTO (PDF # 46-0465) (blue line in Fig. 1b). However, no diffraction peaks belonging to crystalline LLTO can be observed in the XRD pattern of the LLTO thin-film on Si substrate (purple line in Fig. 1b). The diffraction peak at 28.48° should ascribe to the Si substrate. It is reasonable to conclude that the as-deposited LLTO thin-film is amorphous. As shown in Fig. 1c, the as-deposited LLTO thin-film is homogeneous, dense, and without any crystalline grains, which further confirms it is amorphous. The ionic conductivity of α-LLTO thin-film is calculated based on its bulk resistance determined by the intercept on Z_re_ axis of EIS curve (Fig. 1d) and its thickness determined in the side-view SEM image (insert in Fig. 1c). The as-deposited α-LLTO thin-film performs an ionic conductivity of 1.54 × 10^−5^ S/cm at room temperature, which is comparable to the reported values for α-LLTO thin-film solid electrolytes [31, 32]. Additionally, the previous literatures have demonstrated that α-LLTO thin-film solid electrolytes are with excellent chemical and electrochemical stabilities [30]. Therefore, it is potential to construct a highly stable and conductive LCO-electrolyte interface via deposition of α-LLTO on the surface of LCO electrode.

A conformal coating of α-LLTO on LCO electrode can be realized by sputtering deposition. As seen in Fig. 1e, pristine LCO electrode is with a porous microstructure. The surface modification by depositing α-LLTO for 10 min does not change the surface morphology of electrode significantly (Fig. 1f). When the deposition time is extended to 100 min, an overburden can be observed because of the accumulation and agglomeration of α-LLTO particles (Fig. 1g). However, the distribution of La and Ti observed in EDS mapping images (Fig. 1h) suggests that LCO electrode has been evenly and conformally covered by α-LLTO after 10 min deposition. Co and C are also detected by the surface composition analysis, which indicates the thickness of deposited α-LLTO is much thinner than the probing depth of EDS (~ 1 μm). The XRD patterns of LCO electrodes with and without α-LLTO modification are similar and in good agreement with the standard pattern of LCO (PDF#44-0145), even though the deposition time of LLTO is extended to 100 min (green, yellow, pink, red, and black lines in Fig. 1b). This is consistent with the fact that the as-deposited LLTO is in an amorphous form.

It is difficult to directly determine the thickness of LLTO layer on the porous LCO electrode because the surface of porous LiCoO_2_ electrode is rough. However, the thickness of LLTO thin films deposited on a flat substrate with the same processing parameters should be a useful reference. Thus, we deposited LLTO thin films on silicon wafers, and determined the thickness using a profilometer. The thicknesses of LLTO thin films deposited by 10 min, 30 min, 60 min, and 100 min sputtering are 11 nm, 24 nm, 52 nm, and 80 nm, respectively (Figure S1).

**Fig. 2:**
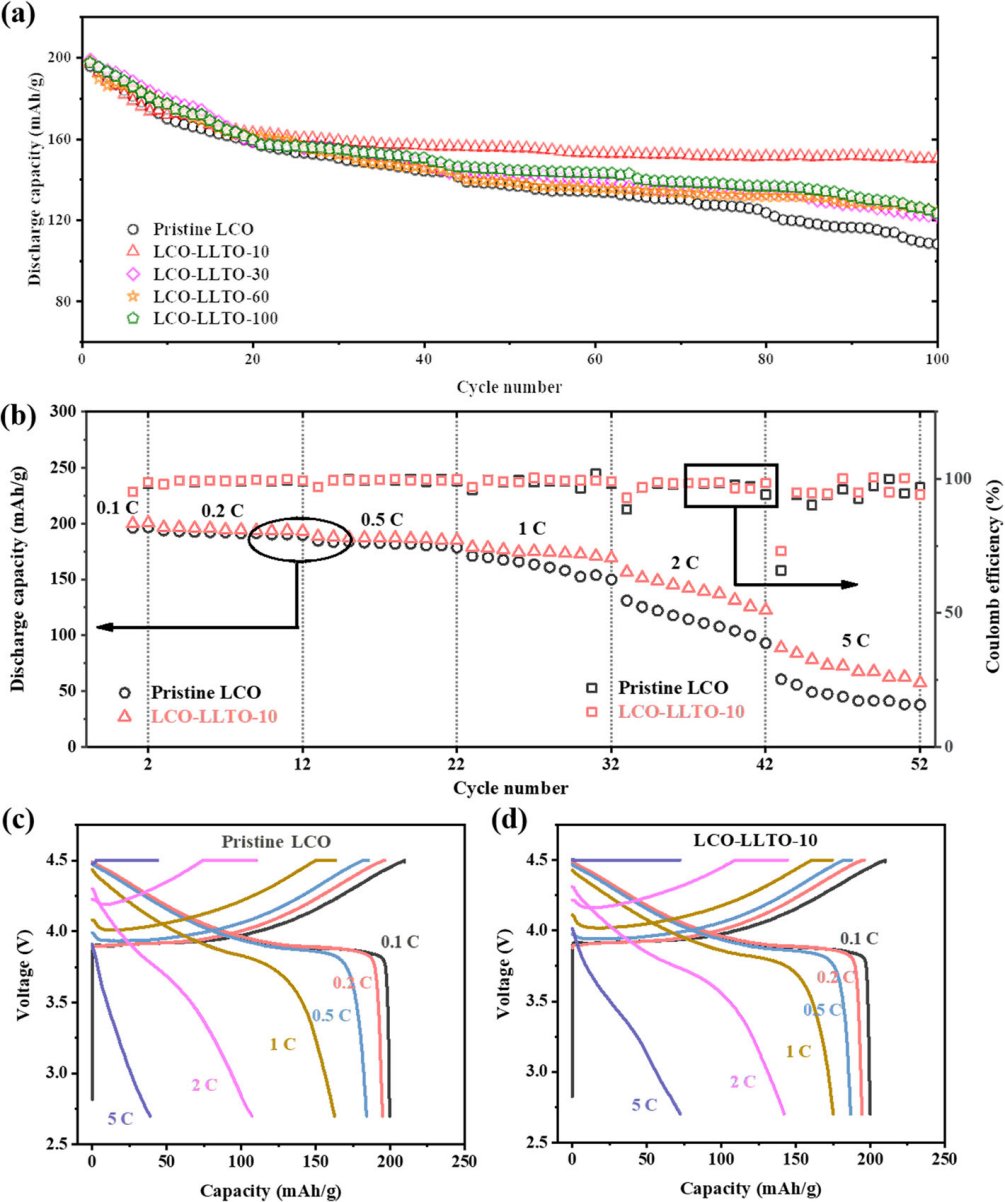
**a** Cycle performances of electrodes based on pristine LCO and that modified by α-LLTO with different deposition time; **b** Rate performances of pristine LCO and LCO-LLTO-10; **c**, **d** Voltage vs. capacity plots of **c** pristine LCO and **d** LCO-LLTO-10 at the different cycling rates

The cycling stability and rate capacity of LCO can be effectively improved via depositing α-LLTO on the made-up electrodes (Fig. 2). The LCO electrodes with and without α-LLTO coating were cycled in the cutoff voltage ranging from 2.75 to 4.5 V vs*.* Li^+^/Li at various cycling rates. In the initial charge/discharge cycles at high voltages, the capacity decay is dominated by irreversible phase transitions and the destruction of crystal structure of LCO [33–37]. Meanwhile, the negative effects of undesired side reactions on the cycling performance of LiCoO_2_ become more and more pronounced in the later charge/discharge cycles because of the sluggish kinetics of the interfacial reactions. The LLTO surface modification mainly addresses the undesired side reactions at the LiCoO_2_-electrolyte interface. Thus, all the samples show similar initial discharge capacities (~ 195 mAh/g) at 0.2 C. And, their capacity retention in the first 20 cycles is closed, as shown in Fig. 2a. In the sequential cycles, the positive effect of the deposited α-LLTO is gradually emerged. After 100 cycles, the discharge capacity of pristine LCO drops to 108 mAh/g. The capacity retention is only 55.4%, comparing with its initial discharge capacity. Meanwhile, LCO-LLTO-10 presents a stable discharge capacity as high as 150 mAh/g after 100 cycles, corresponding to a capacity retention rate of 76.9%. However, a thicker overburden on the surface of porous electrodes will increase the interfacial impedance (Figure S2). The discharge capacities of LCO-LLTO-30, LCO-LLTO-60, and LCO-LLTO-100 after 100 cycles fall in between the pristine LCO and LCO-LLTO-10, which are 122, 124, and 123 mAh/g, respectively. In the later cycles, LCO-LLTO-10 performs a superior capacity retention since, and it may achieve an optimized balance between the cycling stability and charge carrier transport at cathode-electrolyte interface in this study.

It should be noted that a considerable decay of capacity can be observed in Fig. 2a, which should ascribe to the irreversible phase transition and the crystal structure destruction of LCO, although the presented study demonstrated the positive effects of electrode-level LLTO coating on the cycling stability of LCO at high voltages. On the other hand, the efforts of improving the structure stability of LCO cycled at high voltages through foreign elements doping have made significant progress recently [38–40]. It is promising to develop the strategies for promoting the performance of LiCoO_2_ at high voltages based on the synergetic effect of coating and doping.

As cycling rate increases, the positive effect of the α-LLTO modification becomes more and more notable (Fig. 2b). The average discharge capacities of pristine LCO and LCO-LLTO-10 at the different cycling rates are listed in Table 1. After two activating cycles at 0.1 C, the specific capacities of pristine LCO are slightly lower but very closed to that of LCO-LLTO-10 at 0.2 C and 0.5 C. However, LCO-LLTO-10 exhibits remarkably higher capacities than pristine LCO when the cycling rate surpassed 1 C. For example, the discharge capacity of LCO-LLTO-10 reaches 72 mAh/g at 5 C, which is 84.6% higher than that of pristine LCO (39 mAh/g). In addition, the discharge voltage platform of pristine LCO has almost disappeared at 2 C (Fig. 2c), which indicates the aggravated interfacial polarization due to the limited charge transfer kinetics. Meanwhile, LCO-LLTO-10 performs an observable discharge voltage platform (~ 3.76 V) at 2 C (Fig. 2d). The superior rate capacity of LCO-LLTO-10 indicates that the surface modification with a proper α-LLTO deposition time would not only retain the conductive network for Li^+^ and e^−^ in the electrodes, but also somehow enhance its charge transport and/or transfer.

**Table 1:** The average discharge capacities (mAh/g) of pristine LCO and LCO-LLTO-10 at different cycling rates

Cycling Rate	0.1 C	0.2 C	0.5 C	1 C	2 C	5 C
Pristine LCO	200	195	184	163	107	39
LCO-LLTO-10	200	194	187	175	142	72

It is worthy to note that the cells used for testing rate performance did not exhibited the fast capacity decay in the initial 20 cycles (Fig. 2b), which is markedly different from the trend shown in Fig. 2a. It should ascribe to the two activation cycles at 0.1 C before the rate performance testing because these two cycles may help to form a uniform and dense cathode electrolyte interphase (CEI) layer.

**Fig. 3:**
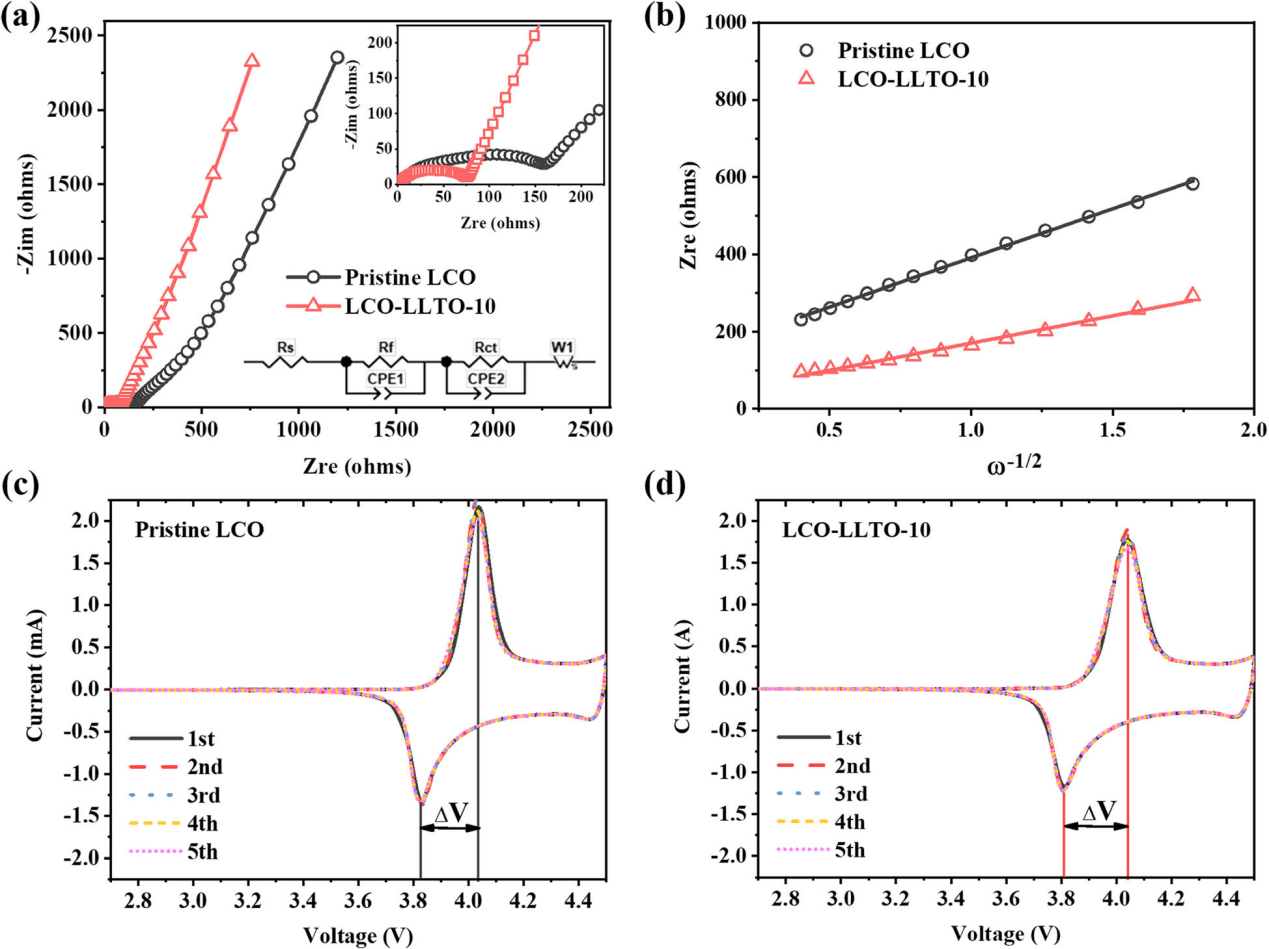
**a** Electrochemical impedance spectra of pristine LCO and LCO-LLTO-10 and the equivalent circuit model; **b** The correlation between Z*re* and ω^−1/2^ of pristine LCO and LCO-LLTO-10; **c**, **d** CV profiles of **c** pristine LCO and **d** LCO-LLTO-10 at a sweeping rate of 0.05 mV/s between 2.75 and 4.5 V

To investigate the effects of the deposited α-LLTO on the conduction in LCO electrodes, EIS measurements of pristine LCO and LCO-LLTO-10 are conducted at room temperature with a cathode-liquid electrolyte-lithium metal configuration (Fig. 3a). The different intercepts on *Z*_*re*_ axis of their EIS curves indicate that tested cell with LCO-LLTO-10 possesses a much smaller total resistance than the counterpart with pristine LCO. The inclined line at low frequency is derived from Warburg impedance, which is related to Li^+^ diffusion within LCO electrodes [41]. The Li^+^ diffusion coefficient ${D}_{Li^{+}}$ can be calculated by Eq. 1 [42]:

1 $$ {D}_{Li^{+}}=0.5\times {\left(\frac{RT}{AC\updelta {F}^2{n}^2}\right)}^2 $$

where *R* is the ideal gas constant (J/(mol*K)), *T* is the Kelvin temperature (K), *A* is the effective electrochemical interfacial area (cm^2^), *n* is the charge number of carrier ions, *F* is the Faraday constant (C/mol)), *C* is the Li^+^ concentration in the unit cell volume (mol/cm^3^), and *δ* is the Warburg coefficient (Ω*(rad/s)^0.5^). The Warburg coefficient *δ* can be determined by Eq. 2 [42]:

2 $$ {Z}_{re}={R}_{\mathrm{total}}+\delta {\omega}^{-1/2} $$

where *Z*_*re*_ is the Warburg impedance, *R*_total_ is the start resistance of the oblique line, and *ω* is the angular frequency corresponding to the impedance sweep frequency *f* (ω=2π*f*). According to the *Z*_*re*_ vs. *ω*^*−1/2*^ plots shown in Fig. 3b, the ${D}_{Li^{+}}$ of LCO-LLTO-10 is determined to be 7.52 × 10^−12^ cm^2^/s, which is much higher than that of pristine LCO (2.32 × 10^−12^ cm^2^/s). This suggests that the bulk ionic conduction in the LCO electrodes is enhanced by the deposited α-LLTO.

The influence of the deposited α-LLTO on the Li^+^ transport across LCO-electrolyte interface should be more pronounced. The measured impedance spectra are further fitted with the equivalent circuit model shown in the inset of Fig. 3a. Here, *R*_*s*_, *R*_*f*_, and *R*_*ct*_ represent the bulk resistance of electrolyte, the resistance of the solid electrolyte interphase on the surface of cathode, and the charge-transfer resistance, respectively. Consequently, LCO-LLTO-10 exhibits a much lower *R*_*ct*_ (40.9 Ω) than pristine LCO (101.8 Ω), indicating the deposited α-LLTO drastically enhanced Li^+^ transport across LCO-electrolyte interface.

It is generally believed that the added interfaces will enhance the difficulties in charge transport and transfer. However, the above EIS analyses suggest that the Li^+^ transport in bulk and charge transfer across interface are both improved via the deposition of α-LLTO on the surface of LCO electrode. This well agrees with the observed excellent rate capacities of LCO-LLTO-10 (Fig. 2c and d), and can be attributed to the following facts. First, the ionic conductivity of deposited α-LLTO is much higher than that of LCO (~ 10^−8^ S/cm) [43]. Second, the sputtering process, which is a physical vapor deposition, enables the deposited α-LLTO to form well-contacted interfaces with LCO particles [19, 20]. Third, the deposited α-LLTO may provide additional Li^+^ transport pathways in the electrodes [19, 42].

It is generally believed that a decreased *R*_*ct*_ would lead to a smaller polarization voltage (ΔV), which is the difference between redox peaks in CV profile. Figure 3 c and d show the CV profiles of pristine LCO and LCO-LLTO-10 for 5 sweeping cycles. The values of ΔV are summarized in Table 2. Unexpectedly, LCO-LLTO-10 exhibits larger ΔV than pristine LCO for the 5 sweeping cycles. The contradiction between the smaller *R*_*ct*_ and larger ΔV for LCO-LLTO-10 can be explained as follows. The experimentally determined *R*_*ct*_ for LCO-LLTO-10 may be derived from the LCO-LLTO interface, while the *R*_*ct*_ for pristine LCO is derived from the interface between LCO and liquid electrolyte. As above mentioned, the physical vapor deposition leads to a well-contacted LCO-LLTO interface, in turn bring about the decreased *R*_*ct*_ for LCO-LLTO-10. Meanwhile, ΔV is derived from the total impedance from liquid electrolyte to LCO. The introduction of α-LLTO may add two interfaces in the LCO-LLTO-electrolyte configuration, which are LCO-LLTO and LLTO-electrolyte interfaces. In addition, the ionic conductivity of α-LLTO is much lower than that of liquid electrolyte, even though it is one of the most conductive solid electrolytes. Thus, larger ΔV are observed in the sample with LCO-LLTO-10. Since the charge transfer between cathode and electrolyte generally is the rate-limiting step, and the Li^+^ transport between α-LLTO solid electrolyte and LiPF_6_-based liquid electrolyte should be relatively fast [42], the better rate capacity and larger ΔV observed for LCO-LLTO-10 is reasonable. More importantly, LCO-LLTO-10 maintains a constant ΔV in the 5 sweeping cycles, while the ΔV for pristine LCO varies and shows an increasing trend (Table 2). This implies that the interface between pristine LCO and liquid electrolyte is continuously degrading, while the LCO-LLTO-electrolyte configuration leads to an excellent interfacial stability.

**Table 2:** The polarization voltage (ΔV) of pristine LCO and LCO-LLTO-10 in 5 cycles

ΔV	ΔV_1st_ (V)	ΔV_2nd_ (V)	ΔV_3rd_ (V)	ΔV_4th_ (V)	ΔV_5th_ (V)
Pristine LCO	0.210	0.206	0.207	0.209	0.210
LCO-LLTO-10	0.230	0.232	0.232	0.232	0.232

As mentioned above, a coating layer of non-conductive materials on individual particles may impede the interfacial charge transfer. Meanwhile, the analysis on *R*_*ct*_ and ΔV indicates that the charge transfer between LCO electrode and liquid electrolyte is promoted by the deposited very thin layer of α-LLTO. Thus, it is reasonable to expect that the cycling stability and rate performance of LiCoO_2_ should be further improved if the individual particles are well-coated by an ultrathin layer of conductive α-LLTO.

**Fig. 4:**
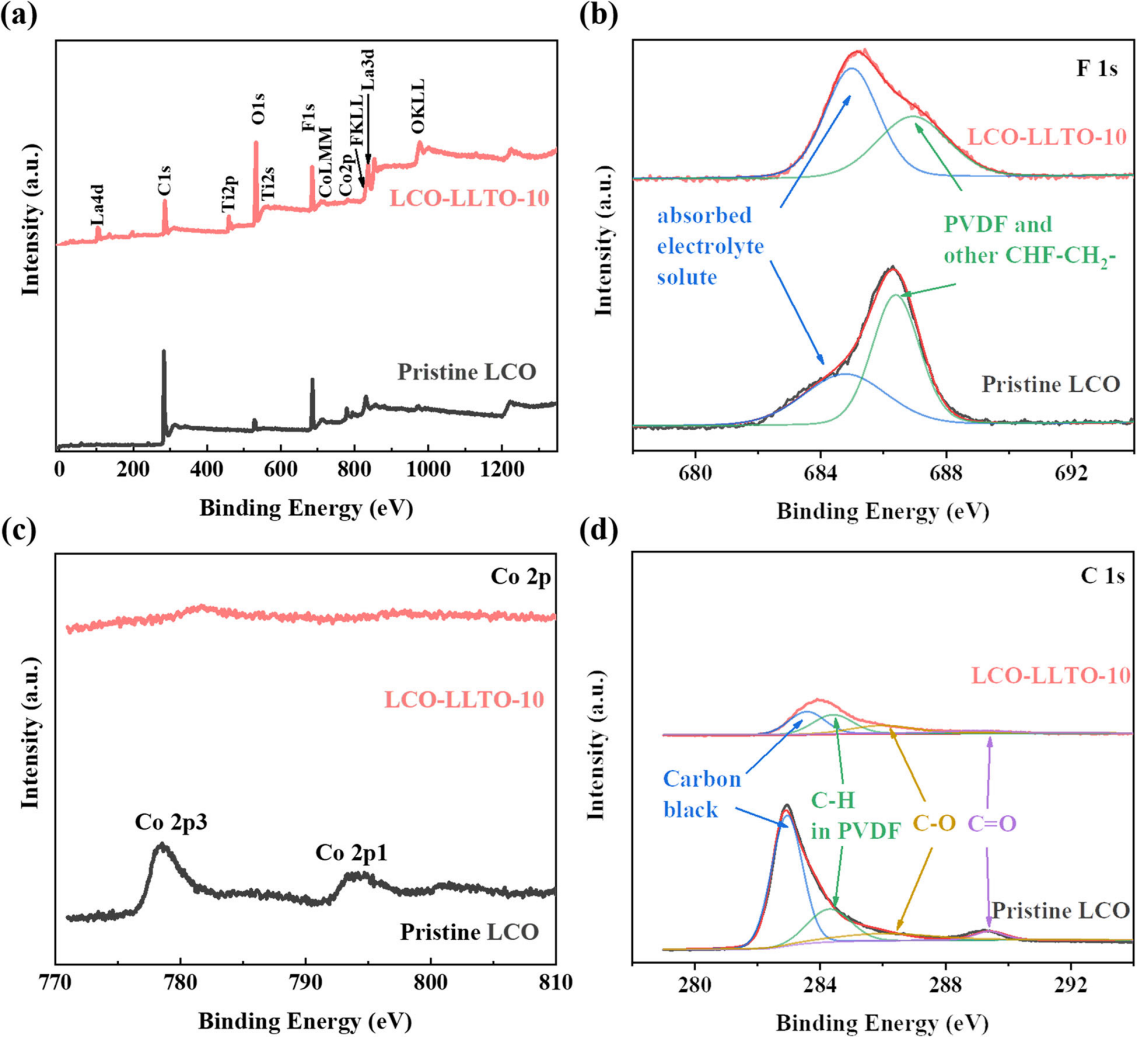
XPS spectra of pristine LCO and LCO-LLTO-10 after 100 cycles. **a** Full spectra, **b** F 1 s spectra, **c** Co 2p spectra, and **d** C 1 s spectra

The potential mechanisms causing the degradation of LCO cycled at high cutoff voltages include, but are not limited to, electrolyte oxidation by delithiated LCO [44], oxygen loss of LCO [45, 46], Co dissolution [47], and HF corrosion at the cathode-electrolyte interface [48]. To reveal how the deposited α-LLTO helps to stabilize the interface, the surface chemistry of the electrodes based on pristine LCO and LCO-LLTO-10 are analyzed by XPS after 100 charge-discharge cycles. The signals of La, Ti, and O are much stronger in the spectrum derived from the cycled LCO-LLTO-10 (Fig. 4a), indicating that La and Ti are not dissolved in the electrolyte during cycling. In addition, the peak at 530 eV, which ascribes to the titanate oxygen in LLTO, is observed in the O 1s spectrum derived from cycled LCO-LLTO-10 (Figure S3). Moreover, the F 1s spectrum only show two peaks derived from PVDF and absorbed liquid electrolyte, and no any peaks related to other fluorides (LaF_3_, TiF_3_, or TiF_4_) are observed (Fig. 4b). These observations suggested that the deposited α-LLTO on the LCO electrode surface is stable during long-term charge-discharge cycling.

For LCO-LLTO-10, LCO particles would be protected from HF corrosion by the deposited α-LLTO, if HF forms due to undesirable side reactions. The stronger O signal derived from LCO-LLTO-10 (Fig. 4a) implies that the oxygen loss of LCO are potentially prevented by the deposited α-LLTO. Additionally, LCO-LLTO-10 presents a much stronger peak ascribed to the absorbed liquid electrolyte solute, comparing with pristine LCO (Fig. 4b). This indicates that the deposited α-LLTO leads to a better wettability of liquid electrolyte on the surface of electrodes or restrains the decomposition of the liquid electrolyte. The peaks corresponding to Co 2p are almost unobserved for LCO-LLTO-10, while that for pristine LCO is obvious (Fig. 4c). The deposited α-LLTO may prevent the diffusion of Co^3+/4+^ to the surface of the electrode and its dissolution into liquid electrolyte. The peaks around 283 eV, 285 eV, 286 eV, and 289 eV observed in C1s spectra (Fig. 4d) are associated with carbon black, PVDF, polyether carbon (O–C–O), and carbonyl group (C=O), respectively [33, 49]. Generally, the polyether carbon and carbonyl group are considered to result from electrolyte decomposition [33, 49]. The absence of C=O peak in the curve derived from LCO-LLTO-10 demonstrates that at least part of the cacoethic side reactions are blocked by the deposited α-LLTO. Based on the above analysis, most of the stability issues of LCO cathode at 4.5 V should be addressed by the deposited α-LLTO.

## Conclusions

In summary, the cycling stability and rate capacity of LCO at high cutoff voltage were improved via depositing α-LLTO on the surface of the made-up electrodes. The effects of the deposited α-LLTO on the LCO-electrolyte interface were studied in details. The results suggest that most of the stability issues of LCO at high cutoff voltage, such as HF corrosion, Co dissolution, and other undesirable side reactions, can be addressed by the deposited α-LLTO. In addition, both of the Li^+^ transport in bulk and the charge transfer across the LCO-electrolyte interface were enhanced through introducing the conductive α-LLTO. With a proper deposition time, the surface modification by α-LLTO enabled LCO steady cycled within 2.75 to 4.5 V vs*.* Li^+^/Li, and performed a reversible capacity of 150 mAh/g at 0.2 C. At high cycling rates, the positive effects of the α-LLTO modification would become more pronounced. The surface modification strategy for LCO presented here provides an encouraging avenue for improving the energy density and cycle life of LIBs.

## Additional File

**Additional file 1: Figure S1.** The thickness of the LLTO thin films with the different deposition time. **Figure S2.** Electrochemical impedance spectra of the as-assembled testing cells with LCO-LLTO-10, LCO-LLTO-30, LCO-LLTO-60, and LCO-LLTO-100. **Figure S3.** O 1s spectrum of LCO-LLTO-10 after 100 cycles.

## Data Availability

The data supporting the conclusions of this article are included within the article and its additional files.

## Abbreviations

LIBs: Lithium ion batteries
LCO: LiCoO_2_
LLTO: Li_3x_La_2/3-x_TiO_3_
α: Amorphous
HF: Hydrofluoric acid
SEM: Scanning electron microscopy
CV: Cyclic voltammetry
EIS: Electrochemical impedance spectroscopy
XRD: X-ray diffraction
EDS: Energy dispersive spectrometer
XPS: X-ray photoelectron spectroscopy
PVDF: Poly(vinylidene fluoride)
EC: Ethylene carbonate
DMC: Dimethyl carbonate
EMC: Ethyl methyl carbonate
CEI: Cathode electrolyte interphase.
