# Supplementary material for: Improved Cycling Stability of LiCoO2 at 4.5 V via Surface Modification of Electrodes with Conductive Amorphous LLTO Thin Film
Source: Nanoscale Res Lett. 2020 May 14;15:110. doi: 10.1186/s11671-020-03335-8 (PMC7225228; doi:10.1186/s11671-020-03335-8)
Supplement: Supplementary file 1 — Additional file 1: Figure S1. The thickness of the LLTO thin films with the different deposition time. Figure S2. Electrochemical impedance spectra of the as-assembled testing cells with LCO-LLTO-10, LCO-LLTO-30, LCO-LLTO-60, and LCO-LLTO-100. Figure S3. O 1s spectrum of LCO-LLTO-10 after 100 cycles. [file 11671_2020_3335_MOESM1_ESM.docx]

Supporting Information for

**Improved cycling stability of LiCoO_2_ at 4.5 V via surface modification of electrodes with conductive amorphous LLTO thin film**

Shipai Song^1^, Xiang Peng^1^, Kai Huang^1^, Hao Zhang^1^, Fang Wu^1^, Yong Xiang^1,2^, and Xiaokun Zhang^1*^

^1^ School of Materials and Energy and ^2^Advanced Energy Research Institute, University of Electronic Science and Technology of China, Chengdu, Sichuan 611731, China

^*^Corresponding author e-mail: zxk@uestc.edu.cn

The LLTO thin films were deposited on the silicon wafer and the thickness of LLTO films were determined using a ME-L (EOPTICS) profilometer.

**Figure S1.** The thickness of the LLTO thin films with the different deposition time.

**Figure S2.** Electrochemical impedance spectra of the as-assembled testing cells with LCO-LLTO-10, LCO-LLTO-30, LCO-LLTO-60, and LCO-LLTO-100.

**Figure S3.** O 1s spectrum of LCO-LLTO-10 after 100 cycles.
